# Anxiety as a mediator between preservice teachers' attitudes toward mathematics teaching and their self-efficacy beliefs

**DOI:** 10.3389/fpsyg.2026.1746400

**Published:** 2026-04-10

**Authors:** Neşe Uygun, Murat Baş

**Affiliations:** 1 Education Faculty, Gaziantep University, Gaziantep, Türkiye; 2 Education Faculty, Kirşehir Ahi Evran University, Kirşehir, Türkiye

**Keywords:** anxiety, attitude, mathematics teaching, mediation analysis, self-efficacy

## Abstract

This study examined the complex relationship between primary school preservice teachers' attitudes toward mathematics teaching and their self-efficacy beliefs, specifically investigating the mediating role of mathematics teaching anxiety. Adopting a quantitative, correlational survey design, data were collected from 360 preservice teachers studying at six universities in Türkiye using a maximum variation sampling strategy. The “Mathematics Teaching Anxiety Scale,” “Attitude Scale Toward Mathematics Teaching,” and “Mathematics Teaching Self-Efficacy Beliefs Scale” were utilized for data collection, with all analyses conducted using Jamovi statistical software. The findings revealed a positive, significant relationship between preservice teachers' attitudes toward mathematics teaching and their self-efficacy perceptions. Conversely, there was a negative, significant relationship between attitudes and anxiety, indicating that more favorable attitudes are associated with lower levels of teaching anxiety. A further negative, significant relationship was established between anxiety and self-efficacy, suggesting that high anxiety is associated with lower teachers' perceived competence. Most critically, mathematics teaching anxiety was found to play a partial mediating role in the statistical association between attitude and self-efficacy. This statistical mediation indicates that positive attitudes are not only directly associated with higher self-efficacy but also indirectly related through their association with lower mathematics teaching anxiety experienced by the preservice teachers. These results underscore the importance of addressing emotional and psychological factors in teacher training. The study emphasizes the critical value of incorporating anxiety-reduction strategies and psychological support into teacher education programs to enhance professional competence and foster positive attitudes, which are vital for long-term student success in mathematics.

## 1 Introduction

Mathematics education stands out as a fundamental discipline in terms of developing individuals' analytical thinking, problem-solving, and critical reasoning skills. Due to its key role in the scientific and technological advancement of modern societies, the quality of mathematics education directly affects student achievement and, consequently, the overall educational level of a country (Sögüt, 2020; Çengel, 2021). In this context, the approaches, skills, and attitudes of primary school teachers—who are one of the fundamental pillars of education—toward mathematics teaching directly shape students‘ perceptions and levels of success in mathematics (Berg et al., 2025; Xenofontos and Andrews, 2020; Ziyanak, 2025; Kaynak, 2024).

In determining teacher qualifications, the psychological processes of preservice teachers toward mathematics teaching are of great importance (Bandura, 1977). Among these processes, attitudes toward mathematics teaching and perceptions of self-efficacy in teaching mathematics are considered critical factors that determine preservice teachers' professional adaptation, teaching strategies, and overall professional motivation (Kuloglu et al., 2024; Demirel and Taşpinar, 2024; San, 2013). Positive attitudes and high perceptions of self-efficacy lay the groundwork for preservice teachers to plan lessons more effectively, motivate students, and be more successful in classroom management (Bandura, 1993). However, mathematics teaching anxiety experienced by preservice teachers during the teaching process may be negatively associated with these positive relationships (Olson and Stoehr, 2019; Lavidas et al., 2023; Ilgiz, 2024; Çay, 2024). The anxiety and tension felt by preservice teachers regarding mathematics teaching may weaken their teaching attitudes and prevent them from feeling competent. In this context, the question of whether mathematics teaching anxiety statistically mediates the relationship between attitudes toward mathematics teaching and self-efficacy perceptions becomes important (Atahan, 2025; Peker, 2016; Gresham, 2018; Wang et al., 2024).

### 1.1 Purpose and significance of the study

The main purpose of this research is to empirically examine the mediating role of mathematics teaching anxiety in the relationship between preservice teachers' attitudes toward mathematics teaching and their self-efficacy beliefs. While previous research has established bivariate links between these constructs, there is a pressing need to clarify the mechanisms through which these variables interact within a single structural framework (Peker, 2016). By adopting a mediation model, this study aims to explain not just if these variables are related, but how anxiety functions as a psychological bridge or barrier in the professional development of future teachers.

The significance of this study lies in its potential to address a critical gap in teacher education and educational psychology. Positive attitudes are often insufficient on their own to guarantee instructional quality; rather, it is the interplay between emotional regulation and perceived competence that determines a teacher's classroom effectiveness (Bandura, 1991; Gresham, 2009; Swars et al., 2009). The findings are expected to provide substantial contributions at both theoretical and practical levels:

By analyzing the interactions among attitude, self-efficacy, and anxiety within an analytical framework, this research expands the application of social cognitive theory in mathematics education (Bandura, 1977; Tatlioglu, 2021). Specifically, revealing the mediating role of anxiety provides a more nuanced understanding of how emotional states are associated with the translation of attitudes into stable self-efficacy beliefs (Cross Francis et al., 2022; Bursal and Paznokas, 2006). This aligns with recent international calls to look beyond cognitive skills and prioritize the “affective domain” in teacher preparation (Hannula et al., 2018).

The results provide a scientific basis for redesigning curricula in teacher training programs. Beyond pedagogical content knowledge, the integration of anxiety-reduction strategies—such as reflective practice and targeted micro-teaching—is vital for fostering professional confidence (Bekdemir, 2010). Furthermore, this study offers a roadmap for educational authorities to implement psychological support systems that mitigate teaching-related stress. Ultimately, cultivating highly self-efficacious and anxiety-free teachers is a prerequisite for enhancing student achievement and long-term mathematical literacy in the global educational landscape.

In conclusion, elucidating the mediating role of mathematics teaching anxiety on the relationship between attitudes and self-efficacy is paramount for optimizing teacher training processes. Ultimately, addressing these psychological mechanisms serves as a strategic pathway toward enhancing student achievement in mathematics and elevating the overall quality of education. This study, therefore, provides a rigorous scientific foundation for developing actionable strategies aimed at professional excellence and educational improvement.

In this research, it is aimed to investigate the mediating role of mathematics teaching anxiety scores in the relationship between preservice teachers' attitudes toward mathematics teaching and their self-efficacy. In line with this main aim, the following questions were sought: In line with this main aim, the following hypotheses were formulated:

- H1: Preservice teachers' attitudes toward mathematics teaching are positively related to their self-efficacy perceptions.
- H2: Preservice teachers' attitudes toward mathematics teaching are negatively related to their anxiety.
- H3: Preservice teachers' anxiety toward mathematics teaching is negatively related to their self-efficacy perceptions.
- H4: Preservice teachers' anxiety toward mathematics teaching mediates the relationship between attitude and self-efficacy.

## 2 Method

In this study, the quantitative research paradigm was adopted. Within the scope of this paradigm, the correlational survey design was employed, which aims to quantitatively describe a population based on a specific sample and examines whether, and to what degree, a relationship exists between two or more quantifiable variables (Creswell, 2002; Fraenkel and Wallen, 2006; Gay et al., 2012). In the present study, this design was used to examine the mediating role of mathematics teaching anxiety in the relationship between preservice teachers' attitudes toward mathematics teaching and their self-efficacy.

In this study, the mediating role of mathematics teaching anxiety in the relationship between participants‘ attitudes toward mathematics teaching and their self-efficacy perceptions regarding mathematics teaching was tested. The mediation model was structured to examine statistical associations among three variables. In the model, participants' attitudes were taken as the independent variable, self-efficacy perception as the dependent variable, and anxiety level as the mediating variable.

**Figure 1:**
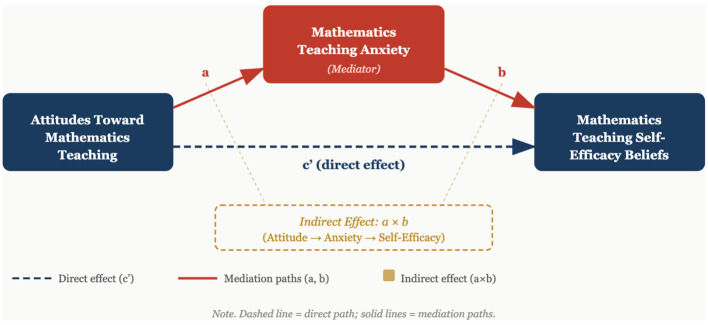
Hypothesized mediation model.

In the structural model, the relationship of attitude with anxiety was examined as path a, the relationship of anxiety with self-efficacy as path b, and the direct statistical relationship of attitude with self-efficacy as path c. Additionally, the indirect association (a × b) and the total association (c) were calculated. The hypothesized relationships and the paths within the mediation model are visually represented in Figure 1.

### 2.1 Population and sample

The population of this research consists of students enrolled in primary school teacher education undergraduate programs during the 2024–2025 academic year. Within the scope of the study, a sample group was formed using the maximum variation sampling method, one of the purposive sampling techniques. Maximum variation sampling aims to reflect the diversity of individuals related to the problem being investigated at the highest level, even with a relatively small sample (Patton, 2002; Cohen et al., 2011). Accordingly, to ensure maximum variation, universities in Turkey with primary school teacher education undergraduate programs were divided into three categories—low, medium, and high—based on their placement base scores in YÖK Atlas data. Two universities from each category were randomly selected. Thus, to reach a sample with maximum variation, data were collected from preservice teachers studying at these six universities during the 2024–2025 academic year. Detailed demographic information regarding the participants' gender, grade level, and university admission score categories is presented in Table 1. Within each selected university, participants were reached through purposive-convenience sampling; preservice teachers enrolled in primary school teacher education programs during the 2024–2025 academic year were invited to participate voluntarily. Ethical approval was obtained from the relevant institutional review board prior to data collection, and all participants were informed about the voluntary nature of the study and the confidentiality of their responses.

**Table 1:** Distribution of participants by demographic characteristics and university categories.

Variable	Category	f	%
Gender	Female	245	68.1
	Male	115	31.9
Grade level	3rd grade	172	47.8
	4th grade	188	52.2
University admission score category	High-scoring universities	128	35.6
	Mid-scoring universities	118	32.8
	Low-scoring universities	114	31.6
University distribution (detail)	University 1 (high)	68	18.9
	University 2 (high)	60	16.7
	University 3 (mid)	62	17.2
	University 4 (mid)	56	15.6
	University 5 (low)	59	16.4
	University 6 (low)	55	15.3
**Total**		**360**	**100.0**

### 2.2 Data collection tools

It should be noted that the Cronbach's alpha coefficient for the attitude scale was 0.69, which falls in the borderline-acceptable range (between 0.60 and 0.70) as identified in the psychometric literature (Zakariya, 2022). Although this value is technically acceptable for exploratory research, it indicates limited internal consistency and should be interpreted with caution. The anxiety (α = 0.92) and self-efficacy (α = 0.85) scales demonstrated good-to-excellent reliability (Göloglu Demir and Çetin, 2012). Permissions for the use of the scales were obtained via email from the developers/adapters of the instruments.

### 2.3 Data analysis

The data obtained in this study were analyzed using the statistical software program Jamovi (The jamovi project, 2024). Various statistical analysis methods were used to test the hypotheses of the research and to examine the relationships between variables. Descriptive statistics (mean, standard deviation, median, minimum, and maximum values) were calculated to determine the general status of preservice teachers' attitudes toward mathematics teaching, self-efficacy, and anxiety levels. To check the assumption of normality, which is one of the basic assumptions of parametric tests, Shapiro-Wilk normality test, skewness and kurtosis coefficients were examined for the continuous variables (attitude, self-efficacy, and anxiety), and related histograms and Q-Q plots were visually evaluated.

To check the normality assumption, which is one of the fundamental assumptions of parametric tests, the Shapiro-Wilk normality test, skewness, and kurtosis coefficients were examined for all continuous variables in the study, namely, mathematics teaching attitude, self-efficacy, and anxiety scores (see Table 2). As a result of the normality analysis, the Shapiro-Wilk test was found to be statistically significant for all three variables (*p* <0.001). This indicates that the distributions statistically differ from the theoretical normal distribution. However, when the skewness and kurtosis values were examined, it was found that all variables had coefficients within the acceptable ±2 range. This suggests that despite the statistically significant deviations, the distributions are quite close to normal. Considering that the sample size (*N* = 360) is robust against violations of the normality assumption in parametric tests and that the skewness/kurtosis values are within acceptable limits, the use of parametric tests was deemed appropriate. Moreover, since the bootstrapping method used for mediation analysis does not require a distributional assumption, it increases the reliability of the results.

**Table 2:** Descriptive statistics and normality test results for continuous variables.

	Anxiety	Attitude	Self-efficacy
*N*	360	360	360
Missing	0	0	0
Mean	63.7	75.2	41.1
Median	66.5	76.0	40.0
Standard deviation	16.0	13.0	7.98
Minimum	24	44	24
Maximum	105	124	70
Skewness	0.0305	0.445	0.879
Std. error skewness	0.129	0.129	0.129
Kurtosis	−0.317	1.03	1.12
Std. error kurtosis	0.256	0.256	0.256
Shapiro-Wilk W	0.965	0.967	0.944
Shapiro-Wilk p	<0.001	<0.001	<0.001

To test Hypotheses H1, H2, and H3, Pearson Correlation Coefficients were calculated to examine the relationships among prospective teachers‘ attitudes toward teaching mathematics, self-efficacy perceptions, and anxiety levels. Finally, to investigate the mediation analysis, a mediation analysis was conducted to test the hypothesis that “prospective teachers' anxiety toward teaching mathematics mediates the relationship between attitude and self-efficacy” (Hayes, 2013). In this analysis, attitude toward teaching mathematics was designated as the independent variable, anxiety about teaching mathematics as the mediator variable, and self-efficacy toward teaching mathematics as the dependent variable. The bootstrapping method with 5,000 resamples was used to test the statistical significance of the mediation analysis. As a result of the analysis, total effect, direct effect, and indirect effect coefficients, along with their significance levels, were examined. For all analyses, the statistical significance level was set at α=0.05. It is important to note that because the present study is based on cross-sectional data, the mediation analysis identifies statistical associations rather than causal relationships. Therefore, the findings should be interpreted with caution regarding causal interpretations (Sage Research Methods, 2024; Maxwell and Cole, 2007).

The raw data supporting the conclusions of this article will be made available by the authors, without undue reservation, to any qualified researcher.

### 2.4 Findings

H1: Preservice teachers' attitudes toward mathematics teaching are positively related to their self-efficacy perceptions.

When Table 3 is examined, a statistically significant and moderate positive relationship was found between preservice teachers' attitudes toward mathematics teaching and their self-efficacy perceptions (*r* = 0.463, *p* < 0.001). This finding indicates that approximately 21.5% of the variance in self-efficacy perceptions can be statistically explained by attitudes toward mathematics teaching, representing a moderate association. Accordingly, preservice teachers who hold more favorable attitudes toward teaching mathematics tend to report higher self-efficacy beliefs in this domain. This result supports H1 and is consistent with Bandura (1997) social cognitive theory, which posits that positive affective orientations toward a task strengthen perceived capability to perform it successfully.

**Table 3:** Correlation analysis results: relationship between attitudes toward mathematics teaching and self-efficacy perceptions.

	Attitude	Self-efficacy
Attitude	Pearson's *r*	0.463^***^
	df	358
	*p*-value	<0.001
Self-efficacy	Pearson's *r*	—
	df	—
	*p*-value	—

^***^p < 0.001.

H2: Preservice teachers' attitudes toward mathematics teaching are negatively related to their anxiety.

When Table 4 is examined, a statistically significant and moderate negative relationship was found between preservice teachers' attitudes toward mathematics teaching and their mathematics teaching anxiety levels (*r* = −0.472, *p* < 0.001). This finding indicates that approximately 22.3% of the variance in mathematics teaching anxiety can be explained by attitudes toward mathematics teaching. Accordingly, preservice teachers who hold more positive attitudes toward teaching mathematics tend to experience lower levels of teaching-related anxiety. This result supports H2 and aligns with previous research demonstrating that favorable orientations toward mathematics reduce uncertainty and fear of failure in instructional contexts (Peker, 2016; Swars et al., 2009).

**Table 4:** Correlation analysis results: relationship between attitudes toward mathematics teaching and anxiety levels.

	Attitude	Anxiety
Attitude	Pearson's *r*	−0.472^***^
	df	358
	*p*-value	<0.001
Anxiety	Pearson's *r*	—
	df	—
	*p*-value	—

^***^p < 0.001.

H3: Preservice teachers' anxiety toward mathematics teaching is negatively related to their self-efficacy perceptions.

When Table 5 is examined, a statistically significant and moderate negative relationship was found between preservice teachers‘ mathematics teaching anxiety levels and their self-efficacy perceptions (*r* = −0.349, *p* < 0.001). This finding indicates that approximately 12.2% of the variance in self-efficacy perceptions can be explained by mathematics teaching anxiety. Accordingly, preservice teachers who experience higher levels of mathematics teaching anxiety tend to report lower self-efficacy beliefs regarding their instructional competence. This result supports H3 and is consistent with Bandura (1997) self-efficacy theory, which identifies physiological and emotional states—including anxiety—as a key source of self-efficacy beliefs. Similarly, Usher and Pajares (2009) noted that elevated anxiety can undermine teachers' confidence in their instructional capabilities.

**Table 5:** Correlation analysis results: relationship between mathematics teaching anxiety and self-efficacy perceptions.

	Anxiety	Self-efficacy
Anxiety	Pearson's *r*	−0.349^***^
	df	358
	*p*-value	<0.001
Self-efficacy	Pearson's *r*	–
	df	–
	*p*-value	–

^***^p < 0.001.

H4: Preservice teachers' anxiety toward mathematics teaching mediates the relationship between attitude and self-efficacy.

The results of the mediation analysis examining the mediating role of anxiety in the relationship between prospective teachers' attitudes toward teaching mathematics, their anxiety levels, and self-efficacy perceptions are presented in Tables 6, 7.

**Table 6:** Mediation analysis results: the mediating role of anxiety in the relationship between attitudes and self-efficacy perceptions of preservice teachers.

Effect	Estimate	SE	95% confidence interval		*Z*	*p*
			Lower	Upper		
Indirect	0.0486	0.0185	0.0151	0.0888	2.62	0.009
Direct	0.2354	0.0406	0.1489	0.3081	5.79	<0.001
Total	0.2840	0.0366	0.2063	0.3508	7.76	<0.001

**Table 7:** Mediation analysis path results for the role of anxiety in the relationship between prospective teachers' attitudes and self-efficacy perceptions.

			Estimate	SE	95% confidence interval		*Z*	*p*
					Lower	Upper		
Attitude	→	Anxiety	−0.5814	0.0654	−0.708	−0.4503	8.89	<0.001
Anxiety	**→**	Self-efficacy	−0.0835	0.0290	−0.142	−0.0279	2.88	0.004
Attitude	**→**	Self-efficacy	0.2354	0.0406	0.1529	0.312	5.81	<0.001

First, when Table 6 is examined, the total association between preservice teachers‘ attitudes toward mathematics teaching and their self-efficacy perceptions was found to be statistically significant (B = 0.2840, *p* < 0.001). The 95% confidence interval for this effect (0.2063–0.3508) does not include zero. This finding indicates that attitude has a positive and significant overall association with self-efficacy perception. The indirect association between attitude and self-efficacy through anxiety was also statistically significant (B = 0.0486, *p* =0.009). Since the calculated 95% bootstrap confidence interval for the indirect effect (0.0151–0.0888) does not include zero, this finding provides strong evidence of the indirect effect. This result suggests that prospective teachers' anxiety about teaching mathematics plays a significant statistical mediating role in the statistical association between attitude and self-efficacy. In other words, changes in attitude are associated with anxiety levels, and these anxiety levels, in turn, relate to self-efficacy perceptions. Even after the anxiety variable was included in the model, the direct association between attitude and self-efficacy remained statistically significant even after anxiety was included in the model (B = 0.2354, *p* < 0.001). The 95% confidence interval for this effect (0.1489–0.3081) does not include zero. This result indicates that attitude is not only associated with self-efficacy through anxiety but also has a direct association independent of anxiety.

Upon examining Table 7, which details each path (relationship) within the mediation model, it was found that prospective teachers' attitudes toward teaching mathematics had a statistically significant negative effect on their anxiety levels. This finding suggests that as attitudes toward mathematics teaching improve, anxiety levels tend to be lower (B = −0.5814, *p* < 0.001). The 95% confidence interval for this relationship (−0.708 to −0.4503) does not include zero. Furthermore, anxiety levels had a statistically significant negative effect on self-efficacy perceptions (B = −0.0835, p =0.004). The confidence interval (−0.142 to −0.0279) does not include zero. This indicates that as mathematics teaching anxiety increases, self-efficacy perceptions tend to be lower (B = −0.0835, p =0.004), consistent with H3 and the broader literature on anxiety and perceived competence. The direct effect of attitude on self-efficacy (B = 0.2354, *p* < 0.001) remained statistically significant even after the anxiety variable was included in the mediation model, as also indicated in Table 6. The 95% confidence interval for this effect (0.1529–0.312) does not include zero.

Consequently, these results indicate that anxiety plays a statistically significant mediating role in the statistical association between attitude and self-efficacy. Therefore, Hypothesis H4 is supported. However, it should be noted that while this mediation is statistically significant, the indirect effect is relatively modest in magnitude [B = 0.049, 95% CI (0.015, 0.089)], and the findings should not be interpreted as indicating strong practical effects. Other variables not included in this model may also contribute meaningfully to preservice teachers‘ self-efficacy. However, since the direct effect remains significant, partial mediation is evident. In this context, prospective teachers' attitudes are associated with self- efficacy both directly and indirectly through their relationship with anxiety levels.

## 3 Discussion

Our study indicates that preservice teachers' positive attitudes toward mathematics teaching are associated with feeling more competent in this domain. This finding aligns with Bandura (1997) Social Cognitive Theory, which posits that positive affective orientations toward a task strengthen perceived capacity to perform it successfully. Consistent with this theoretical framework, Gresham (2009) similarly found that preservice elementary teachers with lower mathematics anxiety reported significantly higher teaching self-efficacy beliefs. More recently, Segarra and Julià (2022) and Wang et al. (2024) similarly reported that preservice teachers with more positive attitudes toward mathematics demonstrated stronger teaching efficacy beliefs, further confirming the link between affective orientation and perceived instructional competence. Furthermore, Cross Francis et al. (2022) demonstrated that psychological and affective constructs play a central role in shaping instructional quality among preservice teachers.

Additionally, it was found that as preservice teachers‘ attitudes toward mathematics teaching improve, their levels of anxiety tend to decrease. This finding supports Peker (2016) and Swars et al. (2009), who established that mathematics teaching anxiety is closely associated with negative attitudes toward the subject. Bekdemir (2010) further argued that negative early experiences in mathematics classrooms contribute to persistent anxiety in preservice teachers, underscoring the importance of cultivating positive attitudes early in teacher preparation programs. Consistent with this, Lavidas et al. (2023) demonstrated that mathematics anxiety and prior mathematics experience were the primary predictors of preservice teachers' attitudes and beliefs toward teaching mathematics, further highlighting the bidirectional relationship between anxiety and attitude in teacher preparation contexts.

Our research also revealed that as preservice teachers' mathematics teaching anxiety increases, their self-efficacy perceptions decrease. This result is consistent with Bandura (1997) self-efficacy theory, which identifies physiological and emotional states—including anxiety—as a primary source of self-efficacy beliefs. Usher and Pajares (2009) similarly noted that elevated anxiety undermines teachers' confidence in their instructional capabilities. Gresham (2018) further demonstrated that mathematics anxiety levels among teachers remained persistent even after 5 years of classroom experience, suggesting that anxiety significantly undermines teachers' long-term sense of professional competence. These converging findings reinforce the view that anxiety functions as a psychological barrier to the development of professional competence.

Most critically, this study indicates that mathematics teaching anxiety plays a partial statistical mediating role in the statistical association between attitude and self-efficacy. Partial mediation indicates that while positive attitudes contribute directly to higher self-efficacy, a meaningful portion of this effect operates indirectly through the reduction of anxiety. This finding is theoretically significant because it reveals that attitude is associated with self-efficacy through two distinct pathways: a direct cognitive-affective route and an indirect route through emotional regulation. This aligns with Hayes (2013), who emphasized the importance of identifying mediating mechanisms rather than relying solely on bivariate relationships. It also overlaps with Philippou and Christou (2003), who highlighted the inhibiting role of mathematics teaching anxiety in the pedagogical development of preservice teachers. Taken together, these results suggest that positive attitudes alone may be insufficient; targeted anxiety-reduction strategies may also be important for strengthening self-efficacy beliefs. Supporting this view, Gresham (2018) demonstrated that mathematics anxiety persists among teachers even after years of classroom experience, underscoring the need to address anxiety at the preservice stage before it becomes entrenched. Similarly, Özben and Kilicoglu (2021) found that self-efficacy, anxiety, and professional beliefs develop as an interconnected system during initial teacher training, further reinforcing the importance of holistic affective interventions in teacher education programs.

These findings carry potential implications for teacher education programs, though the moderate effect sizes observed suggest that other variables not included in this model may also play important roles in shaping preservice teachers' self-efficacy. Beyond developing pedagogical content knowledge, programs should incorporate structured anxiety-reduction strategies such as expanded microteaching—where preservice teachers practice in low-stakes simulated environments before real classroom exposure (Peker, 2009)—as well as reflective journaling and peer observation, which normalize anxiety and foster emotional awareness. Constructive, competence-focused feedback during practicum experiences can further reinforce self-efficacy by providing mastery experiences, one of Bandura (1997) primary sources of efficacy beliefs. Ultimately, cultivating both positive attitudes and low anxiety levels among preservice teachers is a prerequisite for developing the professional confidence necessary to support long-term student success in mathematics.

Regarding model directionality, the present study assumes the theoretical sequence of Attitude → Teaching Anxiety → Self-efficacy, grounded in Social Cognitive Theory (Bandura, 1997) and prior empirical work (Peker, 2016; Lavidas et al., 2023). However, the cross-sectional design cannot rule out alternative or reciprocal orderings. Consistent with Bandura (1986) triadic reciprocal determinism, it is theoretically plausible that lower self-efficacy may heighten teaching anxiety (Hackett and Betz, 1981; Klee et al., 2023), and that accumulated anxious experiences may reshape attitudes toward mathematics teaching (Luo et al., 2024). Empirical support for bidirectional relationships between mathematics anxiety and self-efficacy has been reported in longitudinal research (Ma et al., 2021; Finell et al., 2024). Future longitudinal and experimental designs are therefore needed to establish temporal precedence and to test competing model directions.

## 4 Limitations

Despite its contributions, this study has several limitations that should be considered when interpreting the findings. First, the cross-sectional research design prevents causal inferences. Because all variables were measured at a single point in time, the observed statistical associations do not establish temporal precedence or causal directionality (Maxwell and Cole, 2007). Longitudinal or experimental designs are needed to clarify cause-and-effect relationships among attitudes, teaching anxiety, and self-efficacy. Second, the study relied entirely on self-report measures. All variables—attitudes, anxiety, and self-efficacy—were collected through the same questionnaire administered simultaneously, which increases the risk of common method variance (CMV). CMV can artificially inflate the observed relationships among constructs when data are collected from a single source at one time point (Podsakoff et al., 2003, 2024). Although a formal statistical test for CMV (e.g., Harman's single-factor test or the common latent factor approach) was not conducted in the present study, this limitation should be acknowledged. Future research should employ procedural remedies such as temporal separation of measurements, multi-source data collection, or observer ratings to reduce method bias (Memon et al., 2023). Third, although the reliability of the anxiety (α = 0.92) and self-efficacy (α = 0.85) scales was good to excellent, the attitude scale yielded a borderline Cronbach's alpha of 0.69. This coefficient, while technically acceptable for exploratory research, indicates limited internal consistency and may have attenuated the observed correlations involving attitude (Zakariya, 2022). Future studies should consider revising the attitude scale or using alternative reliability indicators. Fourth, while the mediation findings are statistically significant, the observed effect sizes are moderate (e.g., *r* =0.463 for the attitude–self-efficacy relationship; indirect effect B = 0.049). These moderate magnitudes suggest that other variables not captured in the present model—such as prior teaching experience, mathematics content knowledge, or teacher educator support—likely contribute to the variance in preservice teachers' self-efficacy. The practical significance of the findings should therefore not be overstated. Fifth, the sample was drawn from six universities stratified by admission score using YÖK Atlas data. Although this procedure enhanced the representativeness of the sample across institutional quality levels, the findings may not generalize to preservice teachers in other national or international contexts. Replication studies using diverse samples from different cultural settings are encouraged.

## Data Availability

The raw data supporting the conclusions of this article will be made available by the authors, without undue reservation.
